# Psychological and Behavioral Outcomes From the Perspective of Moral Culture: A Study of College Students

**DOI:** 10.3389/fpsyg.2022.881376

**Published:** 2022-05-09

**Authors:** Wen Hu

**Affiliations:** Office of Development and Planning, Xi’an Aeronautical Institute, Xi’an, China

**Keywords:** symbolization, internalization, moral reasoning, prosocial behavior, psychological wellbeing, moral identity

## Abstract

Although the issue of moral identity and morality is under investigation for the last many years, there is still a need to investigate its role in how it promotes behavior. This study tends to extend this string of literature and attempted to investigate the mediating role of moral reasoning among the elements of moral culture, prosocial behavior, and psychological wellbeing. For this purpose, college students were selected as participants in this study. For this purpose, a two-wave strategy was followed to collect data. In the first wave of questionnaire distribution, 600 college students were contacted to complete the response. A hidden secret code was allotted to each questionnaire to trace the respondent for the second wave. From the distributed 600 questionnaires, 507 questionnaires were received back. At this stage, demographic characteristics and questions related to both the independent variables were asked from the respondents. While in the next wave, 448 questionnaires were received back from the redistributed questionnaires in the second wave. After discarding the incomplete and partially filled questionnaires (17 questionnaires) there were left 431 useable responses. These responses were used to run the tests through structural equation modeling (SEM) through assessment of measurement and structural model. Results indicate that symbolization promotes positive changes in the psychological wellbeing of the students and prosocial behavior of the college students. Moreover, internalization can promote psychological wellbeing. However, the impact of internalization on the prosocial behavior of college students has not been found statistically significant. Moreover, it can be safely concluded that moral reasoning has the potency to mediate the relationship of symbolization and psychological wellbeing as well as prosocial behavior. Moral reasoning also mediates the relationship between internalization and psychological well-being and prosocial behavior.

## Introduction

Recently there is a piece of increasing evidence that the moral identity plays a vital role in the moral functioning by affecting how people might interpret and respond to different situations that involve moral choice and judgment (Cui et al., 2021). The obligation one feels toward engaging in moral actions is related directly to his moral identity *via* his willingness to maintain his self-consistency (Blasi, 1994; Di Blasio et al., 2019). Aquino and Reed (2002) took the social-cognitive perspective and conceptualized the moral identity in the form of an associated network of moral behaviors, goals, and traits that constitute one’s schema of moral character (Lapsley, 2004; Lapsley and Narvaez, 2004). Conformity theory Bernheim (1994) states that individual behavior is affected at large by such social factors as the desire for prestige, acceptance, or popularity. A person might use several possible identities, moral identity is one of them, as a basis for his self-definition as argued by Reed et al. (2007).

People with a high level of Machiavellianism are more likely to get engaged in such behaviors that might lead them to achieve their objectives by any means, legitimate or otherwise (Amir and Malik, 2016). Based on the main value of Machiavellianism, which is “ends justify the means,” such people could be high achievers, and in their way to do so, they are more likely exposed to engage in work counterproductive behavior. As a personality construct, Machiavellianism promotes that one’s manipulative deeds are justified as long as one achieves the desired outcomes. Machiavellians might try to get ahead of colleagues at any cost, moral or not (Hegarty and Sims, 1979; Gunnthorsdottir et al., 2002; Granitz, 2003; Chen and Tang, 2006). Leaders with a high level of Machiavellianism were found engaged in taking unethical decisions for their self-interest as proved by O’Fallon and Butterfield (2013). In addition, several studies support that they involve in unethical opportunistic behavior such as bullying, among other counterproductive behaviors such as cheating, theft, lying, and sabotage. They mainly show high levels of compromised wellbeing, dissatisfaction, and anxiety rather than the lack of guilt feeling for committing deviant actions (Dahling et al., 2009, 2012). But among the affecting factors in this, the literature finds organizational structure and setup, the type of jobs they perform, the career level, skills, and the level of rewards offered to goal-achieving (Jones and Paulhus, 2009). Due to the perspective of high-level Machiavellians and the fact that they are prone to involve in politics with organizations, they tend to look at the moves of others, superiors, peers, and subordinates, as political moves (O’connor and Morrison, 2001; O’Connor et al., 2017). Hence, they tend to use manipulative tactics to be in the spotlight as favorable to others, peers, and superiors (O’Hair and Cody, 1987). In addition to that, they were found highly career-oriented supervisors, taking roles of leadership to influence their co-workers as demonstrated by Kacmar et al. (2004). As moral identity plays the role of a self-regulatory mechanism to propagate moral actions as argued by Reed et al. (2007), people whose position high self-importance on the moral identity get less involved in unethical opportunistic behaviors than do those who place less importance of self-concept (Reed et al., 2007; Burris et al., 2008). The study of Aquino and Reed (2002) demonstrated that there are two dimensions of moral identity, being rooted in the very core of one’s being and as being true to oneself in action. They labeled them as the dimensions of internalization and symbolization. The first (internalization) corresponds to the level to which the set of moral trains is central to the self-concept, whereas the latter (symbolization) corresponds to the level to which such traits are expressed explicitly *via* the individual’s action in a social context.

From the theoretical lenses, this study adds important theoretical insights into the body of knowledge and tends to add new links. First, this study adds to the literature about symbolization and contends that symbolization has the potency to increase the prosocial behavior and psychological wellbeing of college students. This is the contribution of the study from a theoretical perspective. Similarly, this study also extended the literature related to internalization and contends that internalization promotes psychological wellbeing but it does not promote prosocial behavior, which calls for further investigation. Moreover, from a theoretical perspective, this study adds to the body of knowledge related to moral reasoning, this study tested the mediating role of moral reasoning and extends the literature by adding that moral reasoning has the potency to increase the prosocial behavior and psychological well-being under the influence of symbolization and internalization. From the practical point of view, this study advocates that policymakers should promote the senses of symbolization and internalization to increase the pro-social behavior and psychological wellbeing of college students.

## Review of Literature

### Symbolization, Internalization, Prosocial Behavior, and Psychological Wellbeing

Winterich et al. (2013) argue that symbolization represents the level to which a person might tend to convey his moral identity to the external side *via* actions in the world. The one with a high level of symbolization dimension is the one who has the tendency to be involved in explicit activities that might transfer to others the commitment to specific moral ideas and goals. On the other side, when one has a low level of the symbolization dimension of moral identity, he would incline to get involved in such types of public activities. In the model developed by Aquino and Reed (2002), the levels of both dimensions, internalization, and symbolization, do not necessarily correspond to each other, even though there must be some sort of a positive relationship for both of them (Winterich et al., 2013). Rothbard (2001) connotes that intrinsic motivation can be distinguished from absorption as it is specifically task-oriented with a state of positive emotion while absorption is a neutral state. Self-regulation explains the mechanism of linking absorption and attention to engagement theoretically (Lee et al., 2003). Moreover, Kanfer (1990) further clarifies the concept of self-regulation as a process of converting inner impelling cause into behavior stimulus and performance. Resultantly, this order of attention enables an employee to allocate exclusive efforts for on-task and off-task performances related behaviors. According to Ryan and Deci (2001), well-being is optimal functioning and experience. It is parallel to the notion, that individual utility or wellbeing denotes the point of satisfaction where ones’ preferences are satisfied. The concept of wellbeing is based upon two philosophies termed hedonism and eudemonism. Kahneman et al. (2004) relate the hedonic view of wellbeing with positive emotions of happiness and pleasure. While on the other hand, eudemonism is seen as the cultivation of personal resources and their positive contribution, exercising efforts in compliance with one’s inner satisfaction and beliefs (Waterman, 1993). Thus, pleasure and happiness are the focus of hedonic approaches to wellbeing. Diener et al. (1985) state that the most well-known model established for this approach is subjective wellbeing, composed of three main components; the presence of positive affect, satisfaction with life, and the absence of negative affect. The beneficial treatment of the organization such as fairness and supervisor support results in favorable outcomes such as the development of positive emotions and overall satisfaction.

Moreover, the virtue of mindfulness (moral responsibility) assumes a sense of responsibility resulting in symbolization. As advocated by Pandey et al. (2018) in earlier research, mindfulness as moral responsibility has the potency to mitigate the egocentric bias. Moral responsibility can trigger employees to opt a rationalized decision-making, which further can provoke positive behaviors among employees (Jennings et al., 2015). However, moral judgment cannot truly portray how individuals will attempt to address moral issues in the first place (Jennings et al., 2015), but moral judgments can lead toward the betterment of others in the workplace. Moreover, the moral judgment does not automatically lead to moral action; moral actions are influenced by perceptions of individuals and their virtues because these promote moral responsibility. The reason is that when an employee feels that he or she is morally responsible due to the influence of moral principles (Williams and Gantt, 2012).

***H1:*** *Symbolization has a positive relationship with psychological wellbeing.*

***H2:*** *Symbolization has a positive relationship with pro-social behavior.*

***H3:*** *Internalization has a positive relationship with psychological wellbeing.*

***H4:*** *Internalization has a positive relationship with pro-social behavior.*

### Mediating Role of Moral Reasoning

Past studies indicate that moral reasoning is embedded through three phases such as awareness, judgment, intent and behavior, and moral responsibility. The last component, i.e., moral responsibility, tends to deal with individual’s sense of accountability related to the betterment of others. While the second part of this concept, i.e., mindfulness states that the development of virtue is a trait as a result of through interventions. Internalization used to be a focus perception in social theory in psychology, anthropology, sociology, and linked fields. Previous studies indicate that cultural, social, and behavioral systems force an individual to act in a particular way ethic (Chen and Tang, 2006; Strand and Lizardo, 2015). Simply, society constrained people to act in particular (e.g., prosocial) ways. Thus society tends to constrain people from some potential behaviors within organizations *via* expected emotional, ethical, and intellectual forces. Through the expansion of the theory of the cultural system (Kuper, 2003), “internalization,” is the instrument by which people developed all types of social designs (expressive, normative, cognitive, and the like). Thus, it can be a motivating factor to maintain the psychological well-being of individuals across their lifetimes. The reason is that across the life span there come different stages. Thus with the passage of time and age stage the level of psychological wellbeing changes (Olekalns et al., 2014; Strand and Lizardo, 2015). Thus, in this regard, beliefs tend to represent the being “about” various things, events, or people. It is therefore very reasonable to assume that internalization will promote moral reasoning, moral responsibility which will further enhance the prosocial behavior of the employees. We conceptualized that moral reasoning would enhance the employee’s intention to be more inclined toward others’ betterment and would result in positive behaviors at the workplace, i.e., showing pro-social behaviors.

Internalization holds a central position under the domain of cultural theory in the fields of sociology, anthropology, and psychology. Based on the theory of cultural systems, internalization is regarded as a tool and mechanism by which the individuals at the workplace shape and adapt all types of patterns related to culture (Kuper, 2003). These patterns can be normative, cognitive, and expressive. Moreover, internalization is termed as a process by which individuals are encultured. Literature related to internalization states that characteristics of a culture are retained by individuals before and after internalization in a system and then these characteristics become properties of individuals (Kelly et al., 2011; Strand and Lizardo, 2015).

A framework, proposed by Schwartz (2016) explains that ethical decision making based on moral reasoning promotes positive behaviors, which can be termed “retrospection” and this process follows a pattern of activities and during this process, emotion, intuition, reason, and rationalization hold a pivotal position and play a crucial role. Moreover, during this process, both individual and organizational factors play a moderating role, thus moral capacity at an individual level and an environment of the organization help to improve this process.

Thus, an individual who internalizes a belief then tends to show or represent the world the picture based on that belief. Hence based on the above argument it can be drawn that:

***H5:*** *Moral reasoning mediates the relationship between symbolization and psychological wellbeing.*

***H6:*** *Moral reasoning mediates the relationship between symbolization and pro-social behavior.*

***H7:*** *Moral reasoning mediates the relationship between internalization and psychological wellbeing.*

***H8:*** *Moral reasoning mediates the relationship between internalization and pro-social behavior.*

**FIGURE 1:**
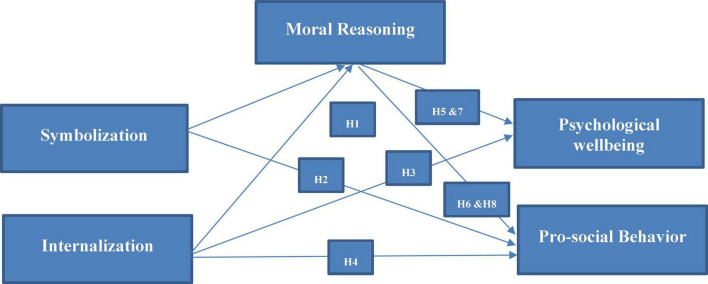
Conceptual framework.

Based on the above literature support and hypothesis development the following framework (see Figure 1) has been established.

## Research Methods

### Study Design

College students were selected as participants in this study. A two-wave strategy was followed to collect data. To set a suitable and well representative sample size, we followed various sample size recommendations based on previous studies. In this regard Krejcie and Morgan (1970), recommendations were kept under consideration. Although a sample size of 384 is considered suitable according to this criteria, however, we have selected a sample size of 600 to be on the safer side. Moreover, according to the general rule of thumb, a benchmark of five to ten respondents against each study construct is considered reasonable and in this case, a sample size of 50 was sufficient. However, we escalated from this sample size recommendation and selected a larger respondent pool. In the first wave of questionnaire distribution, 600 college students were contacted to complete the response. This hidden and secret code was related to the high school of the respondents so that they could be traced easily. At this stage, demographic characteristics and questions related to both the independent variables were asked from the respondents. However, in the second wave questions related to mediating variables and dependent variables were asked from the respondents and at this wave, 448 questionnaires were received back from the redistributed 507 questionnaires. After discarding the incomplete and partially filled questionnaires (17 questionnaires) there were left only 431 useable responses.

This helped us to reduce the potential measurement error of common method biases (Ng and Feldman, 2013; Newman et al., 2015; Feng and Wang, 2019). Moreover, we used reverse coded questions, to potentially restrict the participants from giving monotonic responses. Additionally, we provide a brief purpose statement to the respondents regarding study objectives and their contribution to the literature (Malhotra et al., 2006). These measures helped us to potentially reduce the common method biases.

Respondents were requested to rate their demographic characteristics. From the perspective of gender, female respondents were slightly higher than the male respondents, i.e., 53% female students and 47% male students. While talking about the age group, all the students were under the age limit of 18–25 years indicating an adult population. All the students were enrolled in their undergraduate study courses.

### Measures

Responses were recorded on 5 (5-1) point Likert Scale with a range from strongly agree to strongly disagree where 5 indicates strongly agree and 1 indicates strongly disagree. The first independent variable of this study, i.e., symbolization was assessed based on five items scale. Sample item to measure symbolization includes, “The types of things I do in my spare time (e.g., hobbies).” This scale has been developed by Aquino and Reed (2002) and later on validated by Reed et al. (2007). A second independent variable of this study, internalization is assessed based on four items scale and the sample item for this scale is, “Being someone who has these characteristics is an important part of who I am.” This scale is also developed by Aquino and Reed (2002) and later on validated by Reed et al. (2007). Mediating variable in this study is operationalized based on ten items scale covering the dimension of Conventional Morality. This scale is developed by Christie and Geis (2013), and is known as the Machiavellian IV scale (Mach IV). Previously this scale has been tested and validated by Athota et al. (2009). The dependent variable, prosocial behavior is measured based on pro-sociality in groups. This dimension is most suitable in the context of college students as students tend to form groups in their academic and personal circles. A sample item for this scale includes, “I have shared knowledge with colleagues to help them get ahead.” Initially, this scale is developed by Johnson et al. (1989). However, this scale is validated by researchers in the past, most recently this scale is used by Gotowiec and van Mastrigt (2019). This scale has four items. The last dependent variable (psychological wellbeing) of this study is measured based on five items scale recently used by Akram (2019).

## Results

We have employed the structural equation modeling (SEM) technique for data analysis. SEM was the most suitable technique in this regard to assess the measurement and structural paths. For this purpose, we have used partial least square (PLS) based SEM. Smart PLS 3.9 provides the best measurement parameters in the case of the PLS approach (Avotra et al., 2021; Nawaz et al., 2022). There were also other reasons to opt for Smart PLS software, mainly it deals very well with the non-parametric data and handles complex models very comfortably (Yingfei et al., 2021).

Both measurement and structural models were assessed. First, the measurement model was assessed based on reliability and validity measures. Reliability is assessed through well-established measures, such as alpha, Rho-A, and composite reliability, while validity is assessed through two approaches namely, convergent validity and discriminant validity (Hair et al., 2017). Table 1 in this regard illustrates the reliability and validity measures (convergent validity). First, Cronbach’s alpha was scrutinized for all study constructs and it was observed that all the values were within the acceptable limits, i.e., greater than 0.60. The observed range for alpha values is 0.708–0.882 which indicates a satisfactory level. The lowest value in this regard was observed in the case of psychological wellbeing (0.708) while the heights value of alpha were observed for internalization. Similarly, the second measure of reliability was Rho-A, which also indicates a satisfactory level and all the values of Rho-A were within the threshold limit of Rho-A. The last measure of reliability was composite reliability (CR) in this study. Values of CR in this regard indicate a satisfactory level with an observed range of 0.800–0.927. Therefore all the parameters related to reliability indicate a satisfactory level.

**TABLE 1:** Reliability and validity of the study constructs.

Construct	Item	Outer loadings	VIF	Alpha	Rho-A	Composite reliability	AVE
INT	INT1	0.867	2.256	0.882	0.883	0.927	0.810
	INT2	0.938	3.766				
	INT3	0.893	2.646				
MR	MR10	0.727	2.830	0.870	0.891	0.900	0.565
	MR2	0.766	4.599				
	MR3	0.743	1.799				
	MR4	0.898	4.784				
	MR6	0.589	1.370				
	MR8	0.841	4.296				
	MR9	0.656	3.740				
PSB	PSB1	0.806	3.650	0.832	0.844	0.886	0.661
	PSB2	0.846	4.034				
	PSB3	0.789	1.641				
	PSB4	0.809	1.598				
PWB	PWB1	0.804	1.215	0.708	0.774	0.800	0.504
	PWB2	0.558	1.839				
	PWB3	0.750	1.405				
	PWB4	0.704	2.159				
SMB	SM1	0.834	2.145	0.871	0.876	0.907	0.662
	SM2	0.841	2.286				
	SM3	0.845	3.295				
	SM4	0.817	3.370				
	SM5	0.725	1.844				

*INT, internalization; MR, moral reasoning; PSB, prosocial behavior; PWB, psychological wellbeing; SMB, symbolization.*

Items of the study constructs were scrutinized for poor or weaker outer loading. Items with poor or weaker outer loadings were dropped from analysis to obtain the best parameters. One item from the study constructs internalization was dropped due to poor outer loadings (INT-4). No item was dropped from symbolization and pro-social behavior. While, owing to weaker outer loadings three items (MR-1, MR-5, and MR-7) have been dropped from a total of ten items. Similarly, one item (PWB-5) from the construct psychological wellbeing was removed/dropped from further analysis (Mela and Kopalle, 2002).

Thus outer loading values indicate a sufficient level of indicator reliability/convergent validity. However, some items with weaker outer loadings were retained because the average variance of that construct was within the acceptable limit of equal are greater than 50%. In this regard, MR-6 and MR-9 from the construct moral reasoning while PWB-2 from the construct psychological wellbeing was retained despite weaker or lower outer loading values (Table 1). While another measure of convergent validity in this study is average variance extracted (AVE). Values for AVE were located and it has been observed that all the study constructs are sharing more than 50% variance. A higher level of AVE is observed for the study construct internalization, while lower AVE has been observed in the case of psychological wellbeing.

The second measure of validity is discriminant validity which is assessed based on Fornell and Larcker’s (1981) criterion and HTMT ratio (Hair et al., 2017) (see Tables 2, 3). Smart PLS provides a new method to assess the discriminant validity through the HTMT ratio. The first criterion used in this study is Fornell and Larcker (1981) criterion. Table 3 in this regard illustrates that the square root of AVE of the respective construct is higher than the correlations in the respective column and row. For instance square root of AVE of internalization is 0.90 which is higher than the correlation values in that column, and all the values are less than this square root. A similar pattern is seen in the case of moral reasoning where the square root is higher than the respective correlations in row and column. While the square root of prosocial behavior is also higher than the correlations in row and column. The same pattern is observed for other study constructs, psychological wellbeing and symbolization (Hair et al., 2011). Thus, discriminant validity based on the Fornell–Larcker Criterion is established.

**TABLE 2:** Discriminant validity (Fornell and Larcker, 1981 criterion).

Construct	INT	MR	PSB	PWB	SMB
INT	***0.900***				
MR	0.216	***0.752***			
PSB	0.122	0.377	***0.813***		
PWB	0.377	0.495	0.425	***0.710***	
SMB	0.359	0.285	0.260	0.651	***0.814***

*INT, internalization; MR, moral reasoning; PSB, prosocial behavior; PWB, psychological wellbeing; SMB, symbolization. Bold indicates the relationships.*

**TABLE 3:** Discriminant validity (HTMT).

Construct	INT	MR	PSB	PWB	SMB
INT	–	–	–	–	–
MR	0.225	–	–	–	–
PSB	0.140	0.430	–	–	–
PWB	0.426	0.615	0.540	–	–
SMB	0.410	0.301	0.295	0.658	–

*INT, internalization; MR, moral reasoning; PSB, prosocial behavior; PWB, psychological wellbeing; SMB, symbolization.*

Another measure of discriminant validity in this study is followed based on recommendations by Hair et al. (2014) and HTMT ratios among study constructs were scrutinized for recommended criteria. Table 3 illustrates that HTMT ratios among study constructs depict an acceptable level for both conservative and liberal criteria, because HTMT ratio values are less than 0.85 and 0.90, thus meeting the condition of both liberal and conservative criteria and indicating the establishment of discriminant validity.

**FIGURE 2:**
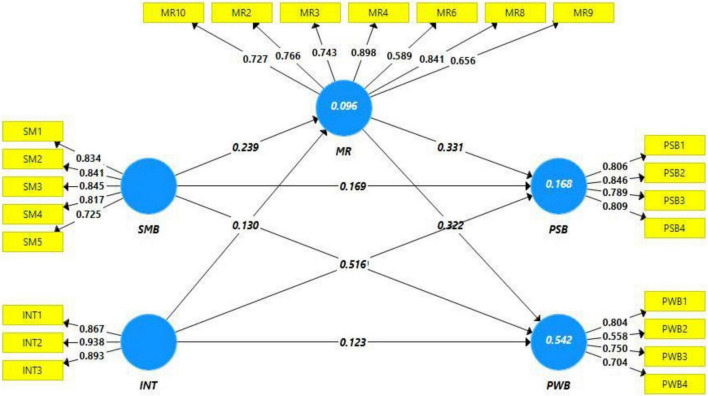
Path estimates and outer loadings.

Model fitness was measured based on effect size while predictive capability was assessed based on the coefficient of determination. First, model fitness based on effect size indicates a satisfactory level of effect size, and values were observed as good. For instance effect size in the case of PWB is observed as 0.204 and 0.479. While talking to a coefficient of determination (predictive accuracy) *R*-square values indicate a good level of predictive accuracy. Figure 2 indicates a 9% change was being observed in moral reasoning, while 16% change was observed in prosocial behavior due to predictors and mediating variables. Similarly, 54% change was observed in psychological wellbeing due to the predictors and mediating variable (moral reasoning) (Hair et al., 2017). In addition to this predictive relevance was also checked and it has been found that the value of *Q*^2^ was higher than the acceptable limit of zero, thus indicating a satisfactory level of predictive relevance (An et al., 2021; Huo et al., 2021).

### Hypotheses Testing

Hypotheses testing have been done based on t and p statistics. Table 4 in this regard provides details regarding direct, indirect, and total paths, while Table 5 discusses the hypotheses testing. In the case of mediation analysis, we have opted to test the significance of indirect effects while simple hypotheses have been tested based on direct path effects. Thus, the first hypothesis (H1) of this study which is related to the impact of symbolization on psychological wellbeing has been found statistically significant as evident by p and t statistics (*t* = 10.684 and *p* = 0.000). Path coefficient in this relationship indicates that one unit change in symbolization will bring 0.516 unit change in the psychological welling among the college students. Moreover, a sign of this path is positive which indicates that symbolization has a positive impact on psychological wellbeing, thus, H1 is accepted. Similarly, H2 of this study is related to the impact of symbolization on the prosocial behavior of college students. Statistical data indicates that statistical parameters for this path are significant (*t* = 3.031 and *p* = 0.002) and depict that one unit change in symbolization will bring 0.169 unit change in prosocial behavior of the college students, thus, H2 is accepted. Similarly, the third hypothesis (H3) of this study is related to the relationship between internalization and psychological wellbeing. Statistical parameters indicate that internalization brings positive change in psychological wellbeing and one unit change in internalization will bring 0.123 unit change in psychological wellbeing. Moreover, this path is statistically significant as evident by p and t statistics (*t* = 3.543 and *p* = 0.000). Thus, H3 of this study is supported by the results. While talking to the fourth hypothesis of this study (H4) it has been observed that the impact of internalization of prosocial behavior of the college students is not statistically significant as evident by the t and p statistics of this path (Table 5). *P*-value escalated in this case from the threshold value, thus, H4 is not supported in this case. The reason for this behavior might be the fact that human beings get irrational (Mazar and Ariely, 2006) when they encounter the opportunity to act opportunistically for their interest, and humans usually are tempted to act in this way (Jensen and Meckling, 1976).

**TABLE 4:** Direct, indirect and total path estimates.

Direct path	Beta	SD	*t*	*p*
INT - > MR	0.130	0.059	2.218	**0.027**
INT - > PSB	−0.010	0.051	0.190	**0.849**
INT - > PWB	0.123	0.035	3.543	**0.000**
MR - > PSB	0.331	0.041	8.120	**0.000**
MR - > PWB	0.322	0.039	8.207	**0.000**
SMB - > MR	0.239	0.043	5.511	**0.000**
SMB - > PSB	0.169	0.056	3.031	**0.002**
SMB - > PWB	0.516	0.048	10.684	**0.000**
Indirect path	**Beta**	**SD**	***t***	***p***
SMB - > MR - > PSB	0.079	0.017	4.534	**0.000**
SMB - > MR - > PWB	0.077	0.017	4.59	**0.000**
INT - > MR - > PSB	0.043	0.021	2.077	**0.038**
INT - > MR - > PWB	0.042	0.020	2.113	**0.035**
Total path	**Beta**	**SD**	***t***	***p***
INT - > PSB	0.033	0.058	0.575	**0.566**
INT - > PWB	0.164	0.040	4.132	**0.000**

*INT, internalization; MR, moral reasoning; PSB, prosocial behavior; PWB, psychological wellbeing; SMB, symbolization. Bold indicates the relationships.*

**TABLE 5:** Hypotheses testing.

Hypotheses		Coefficient (Beta)	S.D	*t*	*p*	Status
H1	SMB - > PWB	0.516	0.048	10.684	0.000	Supported
H2	SMB - > PSB	0.169	0.056	3.031	0.002	Supported
H3	INT - > PWB	0.123	0.035	3.543	0.000	Supported
H4	INT - > PSB	−0.010	0.051	0.190	0.849	Not Supported
**Mediation hypotheses**		**Coefficient (Beta)**	**S.D**	***t***	***p***	**Status**
H5	SMB - > MR - > PWB	0.077	0.017	4.590	0.000	Supported
H6	SMB - > MR - > PSB	0.079	0.017	4.534	0.000	Supported
H7	INT - > MR - > PWB	0.042	0.020	2.113	0.035	Supported
H8	INT - > MR - > PSB	0.043	0.021	2.077	0.038	Supported

*INT, internalization; MR, moral reasoning; PSB, prosocial behavior; PWB, psychological wellbeing; SMB, symbolization.*

While mediation hypotheses have been tested based on indirect effects. The first mediation hypothesis (H5) is related to mediating role of moral reasoning in the relationships of symbolization and psychological wellbeing (SMB MR PWB). This path was assessed based on indirect effect, for which both t and p statistics are significant which indicates that moral reasoning mediates the relationship between symbolization and psychological wellbeing. Thus, H5 is supported by the study results. Similarly, H6 is related to the mediating role of moral reasoning between the relationships of symbolization and prosocial behavior (SMB MR PSB). This path was also assessed based on indirect effect, for which both t and p statistics are significant which indicates that moral reasoning mediates the relationship between symbolization and prosocial behavior. Thus, H6 is supported by the study results.

The other two mediating hypotheses are related to the mediating role of moral reasoning between the relationship of internalization and psychological wellbeing, prosocial behavior. H6 in this regard was assessed based on indirect effect and it has been found moral reasoning mediates the relationship between internalization and psychological wellbeing as evident by t and p statistics related to H7 (indirect path). Thus, H7 is supported. The last and final hypothesis of this study is based on the mediating role of moral reasoning between the relationships of internalization and prosocial behavior. The indirect effect of this path is found statistically significant as evident by p and t statistics (see Table 5). Thus, H8 of this study is supported by the results. In this regard, beliefs tend to represent the being “about” various things, events, or people. It is therefore very reasonable to assume that internalization will promote moral reasoning moral responsibility and which will further enhance the prosocial behavior of the employees. Our conceptualization has been found true that moral reasoning would enhance the employee’s intention to be more inclined toward others’ betterment and would result in positive behaviors at the workplace, i.e., showing pro-social behaviors.

The findings of the study support the relationship which is in line with (Cropanzano et al., 1997). These findings are in connection with the previous studies and indicate that employees may show wellbeing for others when they internalize themselves with the organizational culture (Dahling et al., 2009, 2012).

Internalization holds a central position under the domain of cultural theory in the fields of sociology, anthropology, and psychology. Based on the theory of cultural systems, internalization is regarded as a tool and mechanism by which the individuals at the workplace shape and adapt all types of patterns related to culture (Kuper, 2003). These patterns can be normative, cognitive, and expressive. Moreover, internalization is termed as a process by which individuals are encultured. Literature related to internalization states that characteristics of a culture are retained by individuals before and after internalization in a system and then these characteristics become properties of individuals (Strand and Lizardo, 2015).

In alignment with the previous studies, this study also endorses the findings that organizational structure and setup can affect the employees’ pro-social behaviors (Jones and Paulhus, 2009). The reason might be that due to the perspective of high-level Machiavellians and the fact that they are prone to involve in politics with organizations, they tend to look at the moves of others, superiors, peers, and subordinates, as political moves (O’connor and Morrison, 2001; O’Connor et al., 2017). One more reason might be that they tend to use manipulative tactics to be in the spotlight as favorable to others, peers, and superiors (O’Hair and Cody, 1987). As moral identity plays the role of a self-regulatory mechanism to propagate moral actions as argued by Reed et al. (2007), people who position high self-importance on the moral identity get less involved in unethical opportunistic behaviors than do those who place less importance of self-concept (Reed et al., 2007; Burris et al., 2008). Findings are also endorsed by the theory of the cultural system (Kuper, 2003), which states that “internalization,” is the instrument by which people developed all types of social designs (expressive, normative, cognitive, and the like). So, internalization in culture can be a motivating factor to maintain the psychological well-being of individuals across their lifetimes. The reason is that across the life span there come different stages. Thus with the passage of time and age stage the level of psychological wellbeing changes (Strand and Lizardo, 2015).

## Conclusion

From the empirical findings of this study, it can be concluded that symbolization promotes positive changes in the psychological wellbeing of the students and there is a need to promote symbolization among students to enhance their psychological wellbeing. It will be quite beneficial for the students, which will help them to grow in their future life. Similarly, symbolization can increase the prosocial behavior of college students. This improvement in the prosocial behavior of the students can help them to become active members of society as well as they can become good workers in their professional careers. While talking about the impact of internalization on psychological wellbeing, it can be safely concluded that promoting internalization will increase psychological wellbeing. However, internalization doesn’t need to promote the prosocial behavior of college students. The reason might be that internalization is related to the specific institute while prosocial behavior is not directed toward that specific institute. Moreover, it can be safely concluded that moral reasoning has the potency to mediate the relationship between symbolization and psychological wellbeing. Simply, symbolization promotes moral reasoning which will further increase psychological wellbeing. Similarly, symbolization promotes moral reasoning which further increases prosocial behavior. While in the case of internalization, it can be concluded that moral reasoning mediates the relationship between internalization and psychological wellbeing. Similarly, internalization promotes moral reasoning which further triggers college students to show prosocial behavior. This study’s conceptualization has been found true that moral reasoning would enhance the employee’s intention to be more inclined toward others’ betterment and would result in positive behaviors at the workplace, i.e., showing pro-social behaviors.

## Theoretical and Practical Implications

From the theoretical lenses, this study adds important theoretical insights into the body of knowledge and tends to add new links. First, this study adds to the literature about symbolization and contends that symbolization has the potency to increase the prosocial behavior and psychological wellbeing of college students. This is the contribution of the study from a theoretical perspective. Similarly, this study also extended the literature related to internalization and contends that internalization promotes psychological wellbeing but it does not promote prosocial behavior, which calls for further investigation. Moreover, from a theoretical perspective, this study adds to the body of knowledge related to moral reasoning, this study tested the mediating role of moral reasoning and extends the literature by adding that moral reasoning has the potency to increase the prosocial behavior and psychological well-being under the influence of symbolization and internalization. From the practical point of view, this study advocates that policymakers should promote the senses of symbolization and internalization to increase the pro-social behavior and psychological wellbeing of college students.

## Limitations of the Study

First, this study collected data from college students, and thus their perception can be influenced by so many factors, just as age and gender, while we have not tested the role of demographic characteristics in this study, in the future exploring the role of gender in defining prosocial behavior and psychological wellbeing might bring different results. Similarly, increasing the number of respondents can provide more detailed insights into the future. This study used only one variable as a mediating mechanism, so by adding other mediating or moderating mechanisms, such as their academic performance, the parental profession can provide important results in the future. Moreover, opportunistic behavior can also be taken as moderating phenomenon in future studies because it is stemmed from economics, and is traditionally viewed as a self-interest act (Chohan, 2020), so might provide important insights for future studies. In future studies, leadership style should also be studied in such behaviors as employees might find highly career-oriented supervisors as key players in predicting their helping behaviors (Kacmar et al., 2004). Machiavellianism is more likely to get engaged in such behaviors that might lead them to achieve their objectives by any means, legitimate or otherwise (Amir and Malik, 2016). Such people could be high achievers, and in their way to do so, they are more likely exposed to engage in work counterproductive behavior. Thus in future studies, counterproductive behavior should also be studied along with the personality traits. As a personality construct, Machiavellianism promotes that one’s manipulative deeds are justified as long as one achieves the desired outcomes. Machiavellians might try to get ahead of colleagues at any cost, moral or not (Gunnthorsdottir et al., 2002; Chen and Tang, 2006).

## Data Availability Statement

The original contributions presented in the study are included in the article/supplementary material, further inquiries can be directed to the corresponding author.

## Ethics Statement

The studies involving human participants were reviewed and approved by the Xi’an Aeronautical University, China. The patients/participants provided their written informed consent to participate in this study. The study was conducted in accordance with the Declaration of Helsinki.

## Author Contributions

WH: conceptualization, data collection, and writing the draft.

## Conflict of Interest

The author declares that the research was conducted in the absence of any commercial or financial relationships that could be construed as a potential conflict of interest.

## Publisher’s Note

All claims expressed in this article are solely those of the authors and do not necessarily represent those of their affiliated organizations, or those of the publisher, the editors and the reviewers. Any product that may be evaluated in this article, or claim that may be made by its manufacturer, is not guaranteed or endorsed by the publisher.
